# Feasibility of Group Schema Therapy for Outpatients with Severe Borderline Personality Disorder in Germany: A Pilot Study with Three Year Follow-Up

**DOI:** 10.3389/fpsyg.2016.01851

**Published:** 2016-11-25

**Authors:** Eva Fassbinder, Maren Schuetze, Annika Kranich, Valerija Sipos, Fritz Hohagen, Ida Shaw, Joan Farrell, Arnoud Arntz, Ulrich Schweiger

**Affiliations:** ^1^Department of Psychiatry, University of LuebeckLuebeck, Germany; ^2^Center for Borderline Personality Disorder Treatment and Research, Indiana University - Purdue UniversityIndianapolis, IN, USA; ^3^Department of Clinical Psychology, University of AmsterdamAmsterdam, Netherlands

**Keywords:** borderline personality disorder, schema therapy, group psychotherapy, feasibility studies, outpatients, ambulatory care, personality disorder, cognitive behavior therapy

## Abstract

Borderline Personality Disorder (BPD) is a severe, challenging to treat mental disorder. Schema therapy (ST) as an individual therapy has been proven to be an effective psychological treatment for BPD. A group format of ST (GST) has been developed and evaluated in a randomized controlled trial in the United States and piloted in The Netherlands. These results suggest that GST speeds up and amplifies treatment effects of ST and might reduce delivery costs. However, feasibility in the German health care system and with BPD patients with high BPD severity and comorbidity, and frequent hospitalization, has not been tested to date. We investigated GST in 10 severely impaired, highly comorbid female patients with BPD, that needed frequent hospital admission. Patients received an outpatient ST-treatment program with weekly group and individual sessions for 1 year. Outcome measures including BPD severity, general psychopathology, psychosocial functioning, quality of life, happiness, schemas, and modes, and days of hospitalization were assessed at the start of treatment and 6, 12, and 36 months later with semi-structured interviews and self-report measures. We observed significant decreases in severity of BPD symptoms, general symptom severity, dysfunctional BPD-specific modes and schemas, and days of hospitalization. Functional modes, quality of live and happiness improved. The results of this feasibility study are promising and encourage further implementation of ST outpatient treatment programs even for patients with severe BPD and high hospitalization risk. However, small sample size and the missing of a control group do not allow the generalizability of these findings.

## Introduction

Borderline Personality Disorder (BPD) is a severe mental disorder posing a significant burden on the individual, their families and partners, health care systems and society as a whole. BPD is characterized by a pervasive pattern of instability in interpersonal relationships, self-image, affects, and impulsive behavior (American Psychiatric Association, 2000, 2013). Prevalence is estimated to be 2.7% in non-clinical samples (Trull et al., 2010) and up to 10% in outpatient and 25% in inpatient populations (Widiger and Weissman, 1991; Zimmerman et al., 2005). Without adequate treatment BPD patients utilize a disproportionate amount of inpatient and outpatient psychiatric treatment resources, psychopharmacological treatments, crisis intervention, and other medical services (e.g., surgical care for self-injury, hospitalization after intoxications). The annual costs for untreated BPD in Germany are estimated to be 8.69 billion € annually (Wunsch et al., 2014). Most of the costs are incurred by hospitalizations and day treatment (Wagner et al., 2013).

According to international guidelines psychotherapy is the primary treatment of BPD (American Psychiatric Association, 2001; NICE, 2009). There is no evidence that pharmacological treatment may be effective as a comprehensive treatment for BPD (Stoffers and Lieb, 2015). In the last decades psychotherapeutic treatment of BPD has been significantly improved by the development of BPD-specific methods such as dialectical behavior therapy (DBT), schema therapy (ST), transference-focused psychotherapy (TFP), and mentalization based therapy (MBT) (Stoffers et al., 2012). These specific structured psychotherapies have demonstrated efficacy in reducing BPD-symptoms and general functioning (Stoffers et al., 2012). Besides, ST and DBT have also shown impressive cost reductions of direct and indirect health care costs of approx. 10,000 € per patient per year (van Asselt et al., 2008; Wagner et al., 2014).

However, although these specifically designed treatment programs are (cost)-effective and superior to less structured standard care, implementation and dissemination lag behind. Only a very limited number of patients with BPD receive evidenced-based psychotherapy (Hermens et al., 2011).

Regarding the situation in the Germany there are a number of inpatient treatment units specialized on BPD treatment. There is an urgent need for the creation of specialized outpatient treatment facilities.

Among the BPD-specific methods apt for outpatient programs, ST shows particular promise as it was found to be effective regarding all aspects of BPD and led to significant improvements in quality of life (Jacob and Arntz, 2013; Sempértegui et al., 2013). In the first study, a Dutch multicenter randomized controlled trial (RCT) (*N* = 86), ST was compared to TFP. Both treatments consisted of two individual sessions per week over the course of 3 years. ST had significantly less drop-outs, higher remission rates, a better reduction of BPD-typical symptoms and general psychopathology (Giesen-Bloo et al., 2006a). In addition, ST was more cost-effective (van Asselt et al., 2008). A second Dutch study demonstrated successful implementation of individual ST for patients with BPD in general clinical practice showing comparably good effects with a reduced frequency and duration of therapy (Nadort et al., 2009).

The high number of sessions in individual ST, although cost-effective (van Asselt et al., 2008), causes high delivery costs, and makes it problematic to treat all patients requesting it. Farrell and Shaw developed a group format of ST (Group schema therapy, GST) (Farrell and Shaw, 2012), aiming at a more efficient use of resources. An RCT with 32 female BPD patients, comparing treatment as usual (TAU) alone to 30 sessions of GST and TAU, demonstrated the effectiveness of GST for the treatment of BPD. The GST group showed very low drop-out, high remission rates of BPD, reductions in BPD severity and general psychiatric symptoms as well as improvements in psychosocial functioning with large effect sizes after only 8 months of treatment (Farrell et al., 2009). A second study on GST was a Dutch pilot study with 18 patients treated in a combined format of individual and group ST. This study found large improvements in BPD-symptoms, general psychopathology, schema (mode) measures, quality of life, and happiness (Dickhaut and Arntz, 2013).

These results suggest that specific group factors may catalyze effects of ST and GST may be particularly (cost−) effective. To systemically investigate the clinical effectiveness and cost-effectiveness of GST and to test different formats of delivery of GST (GST only vs. a combination of group and individual ST) a large international multicenter RCT on GST for BPD was planned and is underway now (Wetzelaer et al., 2014).

Since feasibility of GST in the German health care system and with BPD patients with high BPD severity and comorbidity had not been tested yet we conducted a pilot study. This study was performed as a pilot study within the frame of the above mentioned international trial on GST (Netherlands Trial Register, number NTR2392). The major aims were to investigate whether a GST program can be implemented in a German University outpatient treatment center under routine mental health care conditions and whether GST is effective even in patients with high BPD severity, high comorbidity, and a history of frequent hospitalization.

## Methods

### Patients

Ten female patients with a primary diagnosis of BPD with high severity were asked to participate in the study. All patients had multiple hospitalizations and outpatient treatments in the past (including 90% of the patients receiving in- and/or outpatient DBT). All but one also received multiple pharmacological treatments. Despite this intensive treatment they were not in remission and were on a waiting list for patients requiring further treatment. Psychosocial functioning was also severely impaired: None of the patients had paid work or was in an education process, three of the patients had at least a day structure with occupational therapy, two women lived in violent partnerships, and none of the patients had a stable intimate relationship without violence. Two patients lived in supervised group housing. The mean age of the sample was 35 (*SD* 13 years). For the clinical diagnoses we used the Structural Clinical Interview for DSM-IV (SCID I and II) (Fydrich et al., 1997; Wittchen et al., 1997), see Tables 1, 2 for diagnoses. Patients had a very high degree of comorbidity with a mean number of 5.1 (*SD* 1.8) comorbid axis-I-disorders, and 1.8 (*SD* 1.5) comorbid personality disorders. (Note: Some patients had more than one anxiety or addictive disorders.)

**Table 1:** **Frequency of comorbid axis-I-disorders according to SCID-I-Interview at baseline**.

**Axis-I-disorder**	***N***	**%**
Affective disorder	10	100
Anxiety disorders, total	9	90
Posttraumatic stress disorder	7	70
Obsessive-compulsive disorder	4	40
Alcohol/drug dependency/misuse	7	70
Eating disorders	8	80

**Table 2:** **Frequency of comorbid axis-II-disorders according to SCID-II-Interview at baseline**.

**Axis-II-disorder**	***N***	**%**
Schizotypal PD	1	10
Paranoid personality disorder	4	40
Antisocial personality disorder	2	20
Avoidant personality disorder	6	60
Dependent personality disorder	3	30
Obsessive-compulsive personality disorder	2	20

Inclusion criteria were age ≥18 years, a primary diagnosis of BPD according to SCID-II, severity of BPD > 20 in the BPD Severity Index, fourth edition (BPDSI-IV) (Giesen-Bloo et al., 2010; Kröger et al., 2013), an well validated semi-structured clinical interview. Exclusion criteria were Major Psychotic disorder (lifetime diagnosis), intellectual deficit (*IQ* < 80), inadequate communication skills in the German language and acute serious substance dependencies (DSM-IV) requiring clinical detoxification. Exclusion criteria were reduced to a minimum in order to reach a realistic and severely disturbed population representative for the population attending institutions providing maximum psychiatric care in Germany.

### Treatment and therapists

The treatment protocol consisted of a weekly 100 min group session (with all 10 participants in one group) led by two therapists following the GST protocol of Farrell and Shaw (Farrell and Shaw, 2012) combined with weekly 60 min individual ST-sessions following the protocol of Arntz and van Genderen (2009) with the specific aim to support the group sessions. A description of the integration of the two modalities is published in Farrell and Shaw (2012). Therapists met once a week for peer-supervision (60 min) and had weekly supervision with Ida Shaw via Skype (60 min). In addition patients received an optimization of their psychotropic medications (mainly reducing polypharmacy), and in some cases occupational therapy and/or consultations with social workers. This therapy program was offered for one year. After this year treatment could be continued if needed according to patients' preferences and clinical judgment. Five patients continued to receive ST according to a reduced temporal schedule.

A total of eight therapists were involved in the treatments, two of them as group and individual therapists, 6 as individual therapists only. All therapists but one were in their first 5years of psychotherapy training in cognitive behavioral therapy, six of them were already specialized in the treatment of BPD, group therapists had prior experiences with group treatment of BPD. All therapists received ST for BPD training workshops (at least 4 days of training) and GST (at least 3 days of training, for group therapist 6 days of training). The patients in this study were their first patients treated with ST. Treatment integrity was monitored by means of intensive supervision.

Before start of the group sessions 4–6 individual sessions were provided to work out an individual case conceptualization using the ST mode model, and to prepare the group sessions.

Central to all therapeutic interventions was the theoretical model of ST for BPD. ST is based on the idea that aversive childhood experiences, such as physical, sexual or emotional abuse, emotional neglect, and lack of secure attachment, lead to the development of dysfunctional schemas (basic mental representations of the self, relationships to others and the world) and specific emotional-cognitive-behavioral states, so-called modes (Young et al., 2003). The disorder-specific concept for BPD operates almost exclusively using the mode model. The following modes are characteristic for BPD: (a) the vulnerable child mode, (b) the angry/impulsive child mode, (c) the punitive parent mode, (d) the detached protector mode. The healthy modes (healthy adult mode and happy child mode) are usually very weak in BPD.

Therapy goals are to support and comfort the vulnerable child mode, to help the angry/impulsive child mode finding adequate ways to deal with anger and getting needs met in non-impulsive and functional ways, to fight the punitive parent mode, and to reassure the detached protector mode, so that patients can reduce their emotional avoidance and learn healthier strategies to deal with emotions and relationships. A last important goal is to strengthen the healthy modes.

Cognitive, experiential, and behavioral interventions are employed, and the working alliance is characterized by “limited reparenting,” i.e., within professional boundaries therapists behave toward patients like good parents and fulfill some of the needs the patients missed in childhood. This serves as an antidote to traumatic experiences and leads to corrective interpersonal experiences. In GST “limited reparenting” is extended to the whole group and aims at providing a “healthy family”-atmosphere to patients, giving them a sense of belonging and safe connection with others. To create a safe atmosphere a closed group design was chosen and a strong emphasis was placed on setting ground rules for the treatment (e.g., confidentiality, respectful behavior toward each other) and to maintain a specific session structure, which is predictable for patients. Every group session started with a safety imagery (“safety bubble”) to promote safety and bonding, followed by a short opening round on mode awareness. After a discussion of homework in the main working phase a topic relevant for the patients and their stage of therapy was addressed with ST-specific techniques, with a strong emphasis on experiential techniques. After that a new homework assignment was given. To reduce tension at the end of the session an exercise activating the happy child mode was chosen. In the first stage of treatment therapy focused on psychoeducation on BPD, on the BPD relevant modes and how they will be worked with in ST. In the following working phase of treatment the focus was on mode change.

In GST patients can validate, support, confront and advise one another and often experience the reactions and responses of other patients as more “real” than those of therapists. For these reasons, GST might “catalyze” the change processes of ST and lead to faster and deeper changes than individual ST alone (Farrell et al., 2009).

### Clinical outcome measures and assessments

The *primary outcome* was BPD severity assessed with the total score of the *Borderline Personality Disorder Severity Index version IV (BPDSI-IV)*, a semi-structured interview rating all facets of BPD pathology. It assesses frequency and severity of all 9 DSM-IV BPD symptoms over the last 3 months. The total score ranges between 0 and 90. The scores on subscales of the BPDSI-IV provide information on the severity of each of the nine dimensions of BPD. The BPDSI-IV shows excellent psychometric features (Cronbach's alpha = 0.85; interrater reliability 0.99, high validity and sensitivity to change) (Giesen-Bloo et al., 2010; Kröger et al., 2013). A cutoff of 15 points has been empirically found to differ people with BPD from people without BPD; our inclusion criterion of >20 has been used in several studies (Giesen-Bloo et al., 2006a; Nadort et al., 2009; Dickhaut and Arntz, 2013), as it reliable distinguished BPD from non-BPD PDs, and indicates a severe BPD in need of treatment.

*Secondary outcomes* were assessed with the following self-report instruments: The *BPD checklist* is a self-report scale that assesses the subjective burden caused by BPD manifestations. Suitability for use as a treatment outcome measure has been established (Giesen-Bloo et al., 2006b). The *Brief Symptom Inventory (BSI)* was used as an inventory of general psychiatric symptoms. The BSI was developed from its longer parent instrument, the SCL-90-R, and shows similar good psychometric properties (Derogatis and Melisaratos, 1983). General psychosocial functioning and social/occupational functioning was assessed by the *Global Assessment of Functioning (GAF)* and the *Social and Occupational Functioning Assessment Scale (SOFAS)* with a short semi-structured interview, based on axis V of DSM-IV. The GAF is a valid scale of global psychopathology and the SOFAS is a valid measure of social, occupational and interpersonal functioning. Both instruments have excellent interrater reliability (intraclass correlation coefficients > 0.74) (Hilsenroth et al., 2000). Further, the total score of the *Work and Social Adjustment Scale (WSAS)* was used to investigate functional impairment. The WSAS assesses functional impairment at the time of assessment in the domains of work, household, social leisure, private leisure, and family and relationships. It consists of 5 items with a score from 0 to 8. The maximum total score is 40, lower scores indicating higher functioning. The WSAS has shown to be a reliable and valid measure of impaired functioning (Cronbach's alpha of internal scale consistency ranged from 0.70 to 0.94, test-retest correlation was 0.73) (Mundt et al., 2002). Quality of life was assessed by means of two widely used and psychometrically sound self-report instruments: the *World Health Organization Quality of Life questionnaire (WHOQOL-short, total score)* (The WHOQOL Group, 1998), and the *thermometer scale of the EuroQoL* (range 0–100) (Brooks, 1996), which assesses primarily subjective physical health state. Happiness was assessed with the *1-item happiness question* (Abdel-Khalek, 2006) validated in more than 30 countries, with the following response possibilities: (1) completely unhappy; (2) very unhappy; (3) fairly unhappy; (4) neither happy nor unhappy; (5) fairly happy; (6) very happy; (7) completely happy. For a single happiness item high test-retest reliability (*r* = 0.86) and good validity have been reported (Abdel-Khalek, 2006). ST-specific measures were the *Schema Mode Inventory (SMI)* and the *Young Schema Questionnaire—short form (YSQ)*. The SMI consists of 143 items on 16 schema modes and measures the extent to which dysfunctional as well as functional modes are present at the time of assessment. It has an acceptable internal consistency, adequate test-retest reliability and moderate construct validity (Lobbestael et al., 2008, 2010). We used the mean item score of dysfunctional modes and the mean item score of functional modes as outcome. The YSQ is used to measure the presence or absence of 16 core maladaptive schemas at the time of assessment (Young, 1998). The YSQ has an adequate internal consistency and good reliability (Baranoff et al., 2006). The YSQ total score was used as outcome.

All outcome measures were employed before the start of therapy (Baseline), and 3 (M1), 12 (M2), and 36 months (Follow-up) later. All 10 patients completed the baseline assessment. One patient missed M1, 2 patients each missed M2 and follow-up.

Furthermore yearly *days of hospitalization* were assessed in the year before treatment, during treatment and in the first and second year after the treatment interval. Inpatient days in the University Hospital of Lubeck University, which was the main hospital for participating patients, were taken from the internal administration and management program. Moreover, inpatient stays in other clinics were assessed via patient reports.

### Statistical analysis

The statistical analysis was based on the intention-to-treat principle, using all available data from all participants that started the treatment. For all variables except days of hospitalization we used mixed regressions for longitudinal data as this method can deal with missing data and yields more valid estimates of effects than analyzing completers only or using last observation carried forward imputations (Schafer and Graham, 2002). The four assessment points over 36 months of all dependent variables constituted the repeated measure (i.e., time). For the repeated part, a compound symmetry (CS) model was chosen as having the best fit for the covariance structure. For the fixed part, time models were chosen based on visual inspection of the observed means. For most variables, the change in year 1 was approximately linear, followed by a much slower change in the follow-up period of 2 years. In these cases, assessment (0, 1, 2, 3) as a linear covariate represented the time effect. In a minority, the change developed as a linear effect of time, thus represented by year (0, 0.5, 1, 2). In a few cases the change was only obtained in year one after which it stabilized, represented by a “segmented” time effect (0, 0.5, 1, 1). In the happiness ratings we found one outlier with a very deviant pattern of strongly reduced ratings. Analysis of Happiness ratings was therefore done without this outlier. Moreover, for this variable AR1 covariance structure had a superior fit above CS, and was therefore chosen.

Days of hospitalization were analyzed by means of mixed Poisson regression with linear time effect (year).

Besides statistical significance, effect size estimates (ES) according to Cohen (1988) were computed. ES for all assessments except for days of hospitalization are based on the change from Baseline to Follow-up (three years) estimate from mixed regression analysis divided by baseline *SD*. For days of hospitalization ES is based on change over 3 years and baseline *SD* on transformed variables for Poisson regression.

## Results

### Treatment, drop-out and data sets

**Figure 1:**
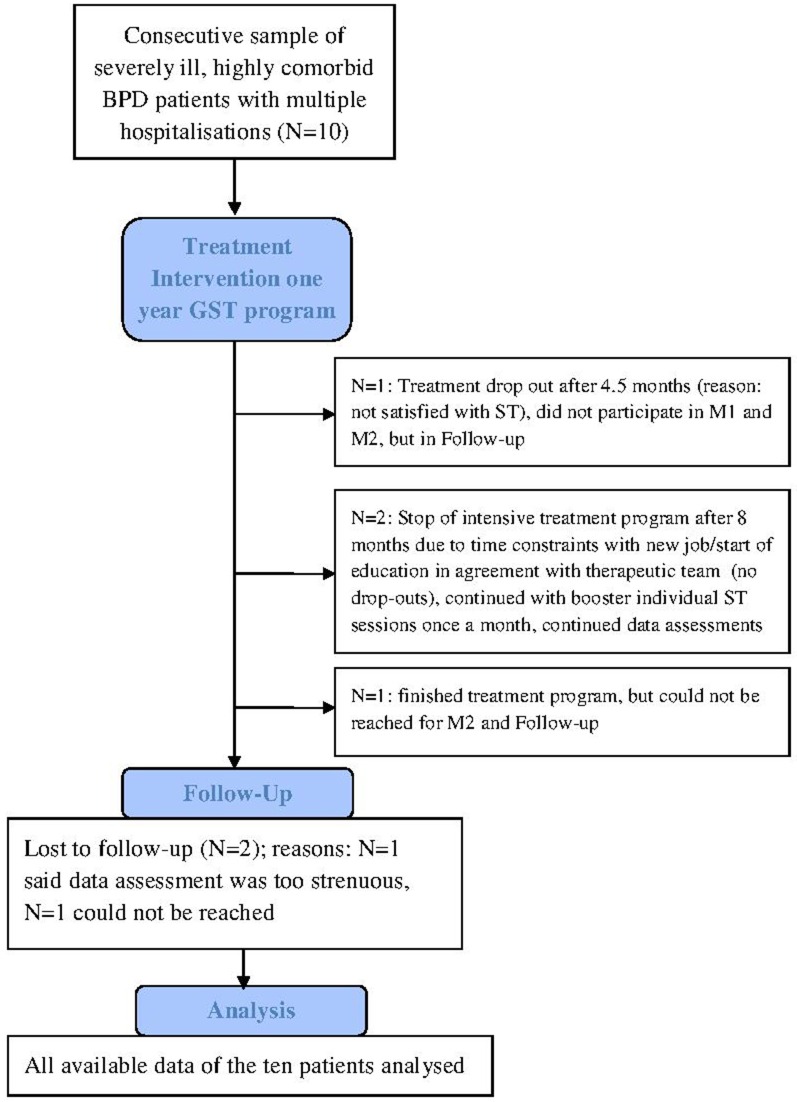
**Consort patient flow chart**.

Figure 1 shows a consort-style patient flow chart. Seven patients completed the treatment. There was one treatment drop-out: One patient decided to stop the treatment program after 4.5 months as she did not feel that ST was the right treatment for her at that time point. This patient did not participate at M1 and M2, but participated in the follow-up-assessment. After 8 months two other patients stopped the treatment program: One patient started professional training and could not organize to attend group sessions anymore, the other patient started a new job and quit the treatment program due to time constraints and as she felt “healthy enough” and this in agreement with the therapeutic team. Both continued with booster sessions once a month and gave their commitment for further data assessment. These two patients are not counted as “drop outs” as stopping the treatment program was in consent with the therapeutic team. After 1 year five of the patients received additional GST and individual therapy sessions in reduced frequency.

### Treatment outcomes

#### Primary outcome: BPD severity

Results from the BPDSI-IV-interview are given in Table 3. A significant reduction in the overall severity of BPD-symptoms with a large ES was obtained. Seven of the nine DSM-IV criteria decreased significantly.

**Table 3:** **Observed means (M) and standard deviations (***SD***) for the four assessment points, results of mixed regression analyses (***F***- and ***p***-value) and estimated effect sizes (ES) for the Borderline Personality Disorder Severity Index, BPDSI (total score and subscales of all nine BPD-criteria)**.

	**Baseline *M* (*SD*)**	**M1 *M* (*SD*)**	**M2 *M* (*SD*)**	**Follow-up *M* (*SD*)**	**Time model^a^**	***F***	**d.f**.	***p***	**ES (Cohen's d)^b^**
BPDSI total score	35.74 (9.34)	29 (11.75)	24.2 (10.16)	19.34 (12.7)	Assessment	15.75	1, 24.72	0.001^*^	1.81
Abandonment	3.17 (1.64)	3.32 (1.9)	2.18 (2.18)	1.39 (1.54)	Assessment	11.84	1, 25.26	0.002^*^	1.25
Unstable relationships	1.78 (1.22)	2.21 (1.4)	1.83 (1.51)	1.22 (0.92)	Year	3.05	1, 25.68	0.093	0.64
Identity disturbance	4.03 (2.62)	3.68 (2.77)	2.62 (2.08)	2.7 (1.8)	Segmented	3.98	1, 24.56	0.057	0.53
Impulsivity	2.24 (1.16)	1.55 (0.85)	1.52 (0.8)	0.94 (0.93)	Year	10.74	1, 25.20	0.003^*^	1.06
Parasuicidality	1.91 (1.09)	1.50 (1.47)	1.12 (0.95)	0.44 (0.5)	Assessment	14.27	1, 25.03	0.001^*^	1.38
Affective instability	8.16 (1.62)	6.44 (2.5)	6.1 (2.6)	5.25 (3.41)	Assessment	13.03	1, 24.37	0.001^*^	1.87
Emptiness	6.73 (2.34)	5.11 (2.69)	4.78 (3.45)	3.47 (3.32)	Assessment	14.52	1, 24.37	0.001^*^	1.42
Anger^c^	2.64 (2.31)	2.02 (1.67)	1.23 (1.73)	1.56 (1.68)	Segmented	4.56	1, 25.6	0.04^*^	0.61
Dissociation & paranoid ideation	5.12 (2.6)	3.2 (2.21)	2.85 (2.4)	2.38 (2.31)	Assessment	15.02	1, 24.75	0.001^*^	1.13

a *Indicates whether time effect was linear over assessments (0,1,2,3) or over years (0,0.5,1,3), or segmented (0, 0.5, 1, 1)*.

b *Based on the baseline to 3-year change from the mixed regression analysis divided by baseline SD*.

c *For the mixed regression analysis, the dependent variable square was root transformed to reduce skewness*.

* *P < 0.05*.

#### Secondary outcomes

Table 4 presents the results for the secondary outcome measures: The subjective burden of BPD manifestations as experienced by the patients (*BPD-checklist; total score*), *general psychiatric symptoms (BSI)*, and *functional impairment (WSAS, total score)* decreased with high effect sizes. *Psychosocial functioning (GAF)* and *social, occupational and interpersonal functioning (SOFAS)* improved with high effect sizes. The improvement of GAF and SOFAS seemed to occur during the treatment interval and to stagnate in the follow-up period. As to quality of live and well-being, the *WHOQol total score* and the *happiness item* increased, while no changes in the *EuroQol thermometer* could be observed. The *ST specific measures* improved with high effect sizes: The mean item score for dysfunctional schema modes decreased, while for the functional modes significant improvement could be obtained. The YSQ total score also decreased.

**Table 4:** **Observed means (M) and standard deviations (***SD***) for the four assessment points, results of mixed regression analyses (***F***- and ***p***-value) and estimated effect sizes (ES) for the Borderline Personality Disorder checklist (BPD, total score), the Brief Symptom Inventory (BSI), the Work and Social Adjustment Scale (WSAS), the Global Assessment of Functioning (GAF) and the Social and Occupational Functioning Assessment Scale (SOFAS), the Schema Mode Inventory (SMI) (mean item score for dysfunctional and functional modes) and the Young Schema Questionaire (YSQ, total score)**.

	**Baseline *M* (*SD*)**	**M1 *M* (*SD*)**	**M2 *M* (*SD*)**	**Follow-upN *M* (*SD*)**	**Time model^a^**	***F***	**d.f**.	***p***	**ES (Cohen's d)^b^**
BPD-checklist	129.11 (27,52)	116.63 (40.44)	109.71 (35.32)	100.38 (43.23)	Assessment	10	1, 22.51	0.004^*^	1.17
BSI	2.35 (0.53)	1.83 (0.93)	2.00 (0.74)	1.50 (1.24)	Assessment	11.19	1, 22.63	0.003^^*^^	1.55
WHOQOL, total score	60.07 (6.44)	66.58 (19.94)	64.09 (15.53)	73.26 (20.26)	year	5.56	1, 23.41	0.027^*^	1.9
EuroQoL-thermometer	44.56 (24)	51.78 (26.32)	47.29 (32.06)	62.43 (32.08)	year	3.24	1, 22.56	0.085	0.71
Happiness item	3.11 (0.78)	3.78 (1.09)	3.14 (1.57)	4 (1.83)	Assessment	4.71	1, 25.96	0.039^*^	1.7
WSAS, total score	24.9 (9.85)	18.56 (14.22)	21.13 (12.02)	14.57 (13.88)	Assessment	19.26	1, 23.21	<0.001^*^	1.09
GAF	36.5 (11.32)	48.67 (18.63)	53.25 (21.95)	51.25 (15.53)	segmented	5.79	1, 25.86	0.024^*^	1.37
SOFAS	45.8 (12.21)	51.78 (14.87)	56.75 (21.37)	53.63 (13.76)	Segmented	5.18	1, 24.70	0.032^*^	0.84
SMI, dysfunctional modes	3.62 (0.55)	3.04 (0.76)	3.02 (0.74)	2.91 (1.03)	Assessment	14.9	1, 21.64	0.001^*^	1.33
SMI, functional modes	2.74 (0.46)	3.33 (0.84)	3.06 (0.69)	3.57 (0.96)	Assessment	8.34	1, 21.93	0.009^*^	1.51
YSQ total	62.2 (9.39)	52.58 (16.28)	53.97 (14.6)	50.37 (17.19)	Assessment	11.75	1, 22.40	0.002^*^	1.34

a *Indicates whether time effect was linear over assessments (0,1,2,3) or over years (0,0.5,1,3), or segmented (0, 0.5, 1, 1)*.

b *Based on the baseline to 3-year change from the mixed regression analysis divided by baseline SD*.

* *P < 0*.

Table 5 shows the days of hospitalizations per year. The inpatient days reduced with a high effect size from an average of 93 days in the year before the treatment program started to 4 days in the second follow-up year.

**Table 5:** **Days of hospitalizations per year in the year before baseline, in year 1–3**.

**Days of hospitalization/year**	**Year before baseline**	**Year 1**	**Year 2**	**Year 3**	***F***	**d.f**.	***p***	**ES^1^ (Cohen's d)**
Original scale (M)	92.9	15.2	22.5	3.8				
Transformed scale (M) (Poisson regression) (s.e.)	4.53 (0.20)	2.72 (0.49)	3.11 (0.40)	1.34 (0.97)	19.14	1, 38	<0.001^*^	5.13

1 *Effect Size Cohen's d based on change over 3 years and baseline SD based on transformed variables from Poisson regression*.

* *P < 0.05*.

## Discussion

The present study supports feasibility of an outpatient ST program with combined group and individual ST under routine mental health care conditions in Germany. Even patients with high BPD severity, a high degree of comorbidity, and frequent hospitalizations can benefit from such a program. Therapists in their first 5 years of cognitive behavioral training outside the centers where ST and GST were developed can learn the method and successfully offer it to patients.

Our findings demonstrate large improvements in BPD-typical symptoms (both in objective and subjective perspective), general psychiatric symptoms, and functional impairment. Psychosocial functioning as well as social and occupational functioning was increased and quality of life (according to the WHOQoL total score) and general happiness improved. However, self-reported evaluation of health state according to the EuroQol thermometer did not improve. A remarkable finding was the massive reduction of inpatient days in the treatment interval and throughout follow-up suggesting that such a treatment program could lead to huge savings in direct health care costs.

The results for the ST-specific measures showed that dysfunctional modes were declining while functional modes improved and that maladaptive schemas reduced. This supports the assumption that the reduction of symptoms was accomplished by the postulated mechanisms of ST.

Of particular note is that patients could profit although 40% of them had one or two comorbid cluster–A personality disorders and 70% had an alcohol or/and drug dependency/misuse, which can be seen as an index of severity of the studied sample and normally leads to exclusion from research trials in BPD psychotherapy. Especially cluster-A personality disorders are often considered as difficult to treat. It should be acknowledged however that the one person with a comorbid schizotypal PD did not profit in a general sense. This specific PD might be a contraindication for group ST, or might need specific adaptations of the program.

The comparison of the results of our pilot study with the other two studies using GST shows high effects of GST on BPD symptoms in all three studies (2.81 for the Borderline Syndrome Index, a different outcome instrument, in the American study vs. 2.71 for the BPDSI total score in the dutch study vs. 1.81 in our pilot study). Treatment drop-out rate was lowest in the American study, 10% in our study and 33.3% in the Dutch study. However, studies can only be compared to a limited extent, since different samples, different doses of treatment and partly different outcome measures have been investigated.

The main limitations of our study are the small sample size and the absence of a control group. Randomized controlled trials with larger samples are needed to further document the efficacy of GST. Moreover, all therapists involved in the study were very engaged and enthusiastic about ST meaning that there might be an allegiance effect even though this was not the center where GST or ST were developed. It also needs to be mentioned that all therapists received extensive training and continuous supervision. The allegiance effect and training might also be responsible for the relatively low drop out rate. With less motivated, less trained and supervised clinicians drop-out might be higher. Higher drop-out may endanger therapy effects for the whole group, especially in a closed group setting.

In this study GST was offered in a closed format. A semi-closed group format would offer more flexibility and would increase implementation possibilities, as not all patients have to start at the same time and new group members can be introduced if patients drop out. However, a semi-closed group format might reduce safety and attachment among group members and therefore interfere with the positive effects of GST. A semi-closed group format has already been developed and tested in a pilot study for inpatients (Reiss et al., 2014). As a semi-closed group format might be helpful for implementation and dissemination in routine health care, the protocol needs to be tested in larger clinical trials also for outpatients.

As far as we know our study is the first study examining outpatient ST for BPD in Germany, and the third study on GST in outpatient care. The results of this pilot study are promising and give us important information of the benefits of structured outpatient treatment programs with GST encouraging further implementation and dissemination.

## Ethics statement

The study was approved by the ethics committee of Lubeck University and has therefore been performed in accordance with the ethical standards laid down in the 1964 Declaration of Helsinki and its later amendments. All subjects gave their written informed consent prior to their inclusion in the study.

## Author contributions

EF, VS, FH, IS, JF, AA, and US planned the study as a pilot project preceding an international multicenter randomized controlled trial on group schema therapy. JF and IS developed the first model of group schema therapy and provided training and supervision. EF, VS, and US adjusted that model to German circumstances and EF, VS, FH, and US implemented the pilot program in the outpatient center of Luebeck university. EF, MS, AK, and US coordinated performance of the study and data assessment. AA provided training and supervision for individual ST. AA, EF, and US did the statistical analyses. EF wrote the first draft of the paper, AA wrote the parts on statistical analysis. All authors edited and revised the manuscript.

## Funding

This pilot study is supported by a grant from the Else-Kröner-Fresenius-Stiftung (P11/10//A90/09). EF obtained funding from the University Luebeck (Einzelprojekt-Forschungsförderung der Sektion Medizin). Funding bodies played no role in the design of the study, in the collection, analysis and interpretation of data, in the writing of the manuscript and in the decision to submit the manuscript for publication.

### Conflict of interest statement

EF, IS, JF, AA, and US give trainings and/or published books on ST or/and GST. MS, AK, VS, and FH declare that the research was conducted in the absence of any commercial or financial relationships that could be construed as a potential conflict of interest.
